# Plants accumulating heavy metals in the Danube River wetlands

**DOI:** 10.1186/2052-336X-11-39

**Published:** 2013-12-20

**Authors:** Marius L Matache, Constantin Marin, Laurentiu Rozylowicz, Alin Tudorache

**Affiliations:** 1 University of Bucharest, Centre for Environmental Research and Impact Studies, Bucharest, Romania; 2 “Emil Racoviţă” Institute of Speleology of the Romanian Academy, Bucharest, Romania

## Abstract

**Background:**

We present herein our results regarding the accumulation of four heavy metals (copper, cadmium, lead, and zinc) in four aquatic species plants (*Ceratophyllum demersum*, *Potamogeton pectinatus*, *Potamogeton lucens*, *Potamogeton perfoliatus*) collected from the Danube River, South-Western part of Romania and their possible use as indicators of aquatic ecosystems pollution with heavy metals.

**Methods:**

Elements concentration from the vegetal material was determined through Inductively Coupled Plasma – Mass Spectrometry.

**Results:**

The species were chosen based on their previous use as bioindicators in aquatic ecosystems and due to the fact they are one of the most frequent aquatic plant species of the Danube River ecosystems within the Iron Gates Natural Park. Highest amounts are recorded for *Ceratophyllum demersum* (3.52 μg/g for Cd; 22.71 μg/g for Cu; 20.06 μg/g for Pb; 104.23 μg/g for Zn). Among the *Potamogeton* species, the highest amounts of heavy metals are recorded in *Potamogeton perfoliatus* (1.88 μg/g for Cd; 13.14 μg/g for Cu; 13.32 μg/g for Pb; 57.96 μg/g for Zn). The sequence for the bioconcentration factors (BCFs) calculated in order to describe the accumulation of the four metals is Cd >> Zn > Pb > Cu. Increase of the zinc concentration determines an increase of the cadmium concentration (Spearman rho=0.40, p=0.02).

**Conclusions:**

Despite the low ambiental levels of heavy metals, the four aquatic plants have the ability to accumulate significant amounts, which make them useful as biological indicators. BCF value for *Ceratophyllum demersum* indicated this species as a cadmium hyperaccumulator.

## Background

Macrophytes have an important ecological role in aquatic ecosystems due to the multiple functions they play in trace elements cycles such as metals removal from water column, suspended matter, riverbed sediments and metals transfer to the food chains [1]. Moreover, the chemical reactions that the plants roots induce in the sediments they grow in increase the sediments capacity to uptake metals [2].

The phytoplankton shows a high accumulation rate of heavy metals entering the aquatic ecosystems [3,4]. Trace elements concentration in plants sometimes reach levels of concentration of six orders of magnitude higher than the environmental one [5]. As a consequence, a number of certain species were found to be useful indicators of the water quality [6] or as solutions for wastewater treatment through phytoremediation processes [7,8], because native plants have the advantage of adapting to the stress conditions of the target pollutants [9].

Metal uptake by vegetation can be element-, plant species- [10,11] and plant tissue- specific [12,13]. For example, *Ceratophyllum demersum* was identified as a hyperaccumulator species for cadmium [14], *Salvinia minima* has the capacity to remove lead from aqueous solutions [15], whilst *Cladophora sp*. is able to hyperaccumulate arsenic [16]. The capacity of plants to hyperaccumulate trace elements is influenced by the presence of humic substances or other chelating substances [17,18], temperature and salinity [19] or amount of metal released through leaves [20].

The Danube River is a transboundary water body suffering a high anthropogenic pressure along its course [21] and a peak of sediments contamination with heavy metals in the Iron Gates area [22].

Little information is available on the amount of heavy metals in aquatic plant species from the Danube River ecosystems in Romania and is limited to the Danube Delta [23,24]. A broad study that includes our target area of interest was performed on the Serbian side of the Danube River with *Ceratophyllum demersum* and *Potamogeton* genus samples collected from 36 sites on a 573 km length sector [25].

Detailed studies regarding the heavy metals levels of concentration in sediments have been performed on the analysed sector, since significant pollution sources are located in the area for more than four decades [26]. They represented the starting point in our survey for targeting certain elements. Our research aims are: 1) to investigate the amount of heavy metals in water and four plant species (*Ceratophyllum demersum*, *Potamogeton pectinatus*, *Potamogeton perfoliatus*, *Potamogeton lucens*) collected from the Danube River wetlands within the Iron Gates Natural Park in South-Western Romania and 2) to discuss the opportunity of using these aforementioned plant species as indicators of the aquatic ecosystems pollution level with heavy metals.

## Methods

We collected water samples from 3 locations (Bazias, Divici, Coronini) in the analysed area, performing four sampling campaigns, one corresponding to each season. Samples were collected in high density polypropylene bottles and nitric acid was immediately added after collection to avoid analytes loss.

A number of 35 plant samples were collected between December 2010 and July 2012, during different vegetation seasons, in order to obtain accurate data set. The plant species under consideration were *Ceratophyllum demersum* (N=12 samples), *Potamogeton perfoliatus* (N=14), *Potamogeton lucens* (N=2) and *Potamogeton pectinatus* (N=7). For the *Potamogeton* genus, the collected sample was composed mainly from leaves and stems, but not roots. The epiphytic plants were removed before storing the samples in plastic bags and frozen at −20°C prior to chemical analysis. For each location, a sample was composed of three plants of the same species collected and pooled into a uniform sample. Samples were superficially washed with distilled water in order to remove particulate matters and then rinsed with ultrapure water (TKA Ultra Pure System GenPure, resistivity 18.2 MΩ×cm).

The samples were dried at 105 °C for 72 hours and then finely grinded prior to digestion. Sample digestion (approximately 2 grams) was performed in an open system with 8 mL of ultrapure concentrated HNO_3_ in a Teflon jar and on an electric plate until the complete dissolution of the organic matrix. The remaining acid extract was diluted with 5 mL of ultrapure water and the resulting solution was filtered on a filter paper with medium porosity (Whatman no 54) and collected in a 25 mL volumetric flask that was brought to constant volume with ultrapure water.

We have determined the elements concentration from the vegetal material using a NexION 300S (PerkinElemer, Shelton, CT, USA) ICP-MS instrument (Serial No. 81SN2032001), equipped with a S10 Autosampler. The operating parameters of the instrument are summarized in Table 1. All the solutions were prepared with ultrapure water (LaboStar™ TWF UV7 Ultrapure Water System, electric resistance 18.2 MΩ×cm).

**Table 1:** **The instrumental settings for the ICP**-**MS**

Spray chamber:	Quartz Cyclonic
Nebulizer:	Meinhard® Concentric Quartz Type A0.5
Torch:	Standard Quartz
Sampler Cone:	Platinum
Skimmer Cone:	Platinum
Plasma gas flow:	18 L min^-1^
Auxiliary gas flow:	1.20 L min^-1^
Nebulizer gas flow:	1.04 L min^-1^
ICP RF power:	1550 W
Pulse voltage:	900 V
Analog stage voltage:	−1637 V
KED mode cell entrance voltage:	−4 V
KED mode cell exit voltage:	−38 V
KED mode axial field voltage:	475 V

**Figure 1:**
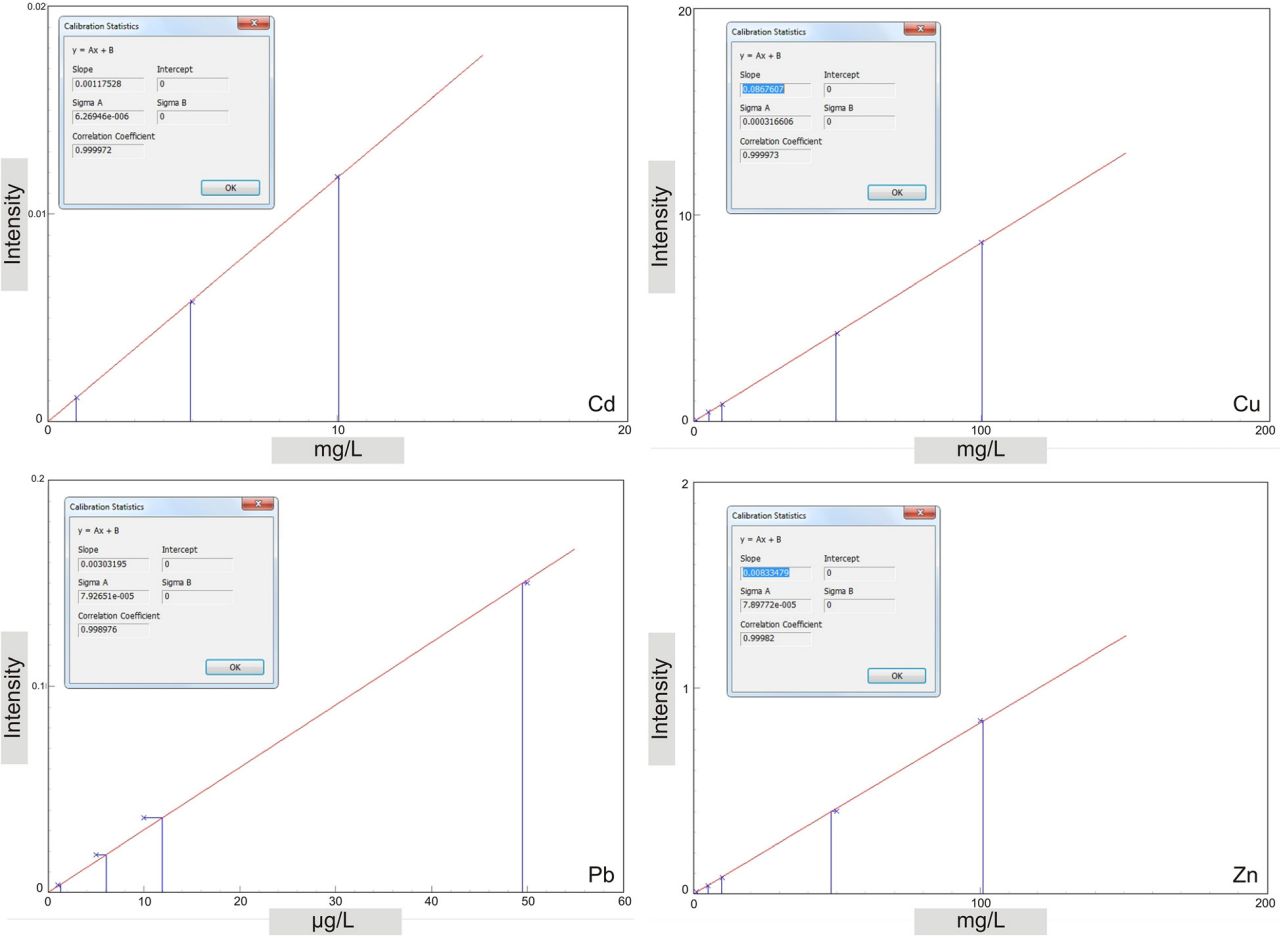
Calibration curves for each element.

The calibration curves (Figure 1) were determined starting from a Multi Element Calibration Standard 3 solution provided by PerkinElmer (Part No. N9300233, Lot No. CL7-173YPY1). The solution contains 30 elements, each of them in a concentration of 10 mg/L. The working solutions for internal standard were prepared starting from the Internal Standard Mix, PerkinElmer Part No. N9303833, Lot No. CL43-46AS, (10 mg/L, Bi, Ge, In, ^6^Li, Sc, Tb, Y) solution. Quality Control (QC), checking and Continuing Calibration Verification (CCV) solutions preparation used CertiPUR® Reference Material, ICP Multielement Standard Solution XXI (Merck, Cat. No. 109498, Lot No. HC 142053). HNO_3_ 60% Ultrapur® (Merck, Cat. No. 101518) was used for the vegetal material digestion, as well as the blanks preparation and washing solutions.

The solutions resulted from samples digestion were diluted 1:5, and the internal standard was added to the samples in order for each used element to reach a concentration of 20 μg/L. These were ^74^Ge for ^63^Cu and ^66^Zn; ^115^In for ^111^Cd; ^209^Bi for ^208^Pb. The instrument was set to work for all elements in kinetic energy discrimination (KED) mode, with He gas flow rate of 3.6 and 4.5 mL/min. For each element, the calibration curves (Figure 1) were achieved using five standard concentrations (0, 0.1, 1, 10 and 100 μg/L) in 1% nitric acid solutions.

QC samples with concentrations of 0.5, 5 and 50 μg/L were analysed after calibration and at the end of samples determination. A 10 μg/L multielement standard was inserted in the analytical sequence every five samples for CCV. Three replicas were recorded for each determination. All analytes were determined with a dwell time of 50 ms per AMU and 1000 ms integration time. Element concentrations were expressed as mg/kg (ppm) in the original samples.

Instrument detection limit (IDL) with 95% confidence level was established for each analyte by the three-time multiplication of the standard deviation obtained from the analysis of ten runs of blank samples in the same day with the determinations. We have obtained the following IDLs: ^63^Cu, 0.015 μg/kg; ^66^Zn, 0.049 μg/kg; ^111^Cd, 0.019 μg/kg; ^208^Pb, 0.074 μg/kg.

Method detection limits (MDL) were estimated to be: ^63^Cu, 0.129 μg/kg; ^66^Zn, 0.264 μg/kg; ^111^Cd, 0.192 μg/kg; ^208^Pb, 0.143 μg/kg. For quality assurance of plant analysis for metals, recovery tests were performed by analysing spiked real samples. Average recoveries (n = 5) were: ^63^Cu, 100%; ^66^Zn, 109%; ^111^Cd, 88%; ^208^Pb, 91.

## Results and discussion

### Statistics

We analysed the concentrations of heavy metals using descriptive statistics and non-parametric analysis using Mintab 16 (Minitab Inc.). Data normality was tested using Shapiro-Wilk test. For all statistical analyses we used α = 0.05. First, we tested the differences between the concentrations of heavy metals in the four analysed species (i.e. *Ceratophyllum demersum*, *Potamogeton pectinatus*, *Potamogeton perfoliatus*, *Potamogeton lucens*) using ANOVA non-parametric Kruskal-Wallis test. Further, we grouped the species on genus and tested the differences of heavy metals concentration between *Ceratophyllum* and *Potamogeton* genera using Mann–Whitney test. In order to test whether the heavy metals concentration from all the analysed samples shows sampling site differences on the Bazias – Coronini sector, we used the same ANOVA non-parametric Kruskal-Wallis test. When Kruskal-Wallis tests indicated significant differences between the analysed groups, the analysis was further developed using Dwass-Steel-Critchlow-Fligner test for multiple comparisons. Finally, we tested the associations between Zn and Cd elements concentration using Spearman rank correlation [27].

### Metals concentration in water and plant samples

The concentration of heavy metals in the water samples we analysed is much lower than the Romanian standards for freshwater quality [28]. The concentration values (given as average of the results from the four campaigns) are shown in Table 2. Lead level is the only one that may generate a possible threat to the water quality in Coronini sampling point.

**Table 2:** **Metal concentration** (**average** ± **1 standard deviation**) **in water samples**

**Sampling point**	**Cadmium (μg/L)**	**Copper (μg/L)**	**Lead (μg/L)**	**Zinc (μg/L)**
Baziaş	0.002 ± 0.004	1.89 ± 0.04	SLD	1.82 ± 3.49
Divici	SLD	3.17 ± 1.86	SLD	1.35 ± 1.89
Coronini	0.008 ± 0.006	1.46 ± 0.59	2.76 ± 3.22	0.78 ± 0.86
Romanian standard	1	20	5	100

Maximum admitted concentrations by the Romanian Standard for freshwater are included.

As a general observation, most of the measured concentrations show a great dispersion (high values of the standard deviation - see Tables 2, 3 and 4). This can be a consequence of the fact that the samples are collected over a longer period of time (18 months) and during different vegetation seasons. Annual balance of precipitation, extreme river conditions (flooding in the spring, drought in the summer) and biological activity of different intensity can also influence the metal uptake rate. For the cadmium in water samples, the high dispersion of data can be determined by the metal low level of concentration (the average value is 2 ng/L). Because of the high values of standard deviation, the data follow a non-normal distribution. Thus, we have chosen to analyse the data with non-parametric statistics.

**Table 3:** **Metals concentrations** (**average** ± **1 standard deviation**) **in aquatic plant samples**

**Plant species**	**Cadmium (μg/g)**	**Copper (μg/g)**	**Lead (μg/g)**	**Zinc (μg/g)**
*C*. *demersum*	3.52±2.07	22.71±21.08	20.06±8.68	104.23±102.76
*P*. *pectinatus*	0.64±0.91	6.24±2.99	6.63±6,47	16.38±8.04
*P*. *perfoliatus*	1.88±1.49	13.14±4.87	13.32±19.88	57.96±95.39
*P*. *lucens*	0.97±0.59	9.80±2.08	1.51±1.05	15.63±5.65

**Table 4:** **Metals concentrations** (**average** ± **1 standard deviation**) in ***Ceratophyllum* and *Potamogeton * genera**

**Plant genera**	**Cadmium (μg/g)**	**Copper (μg/g)**	**Lead (μg/g)**	**Zinc (μg/g)**
*Ceratophyllum*	3.52±2.08	22.71±21.08	20.06±8.68	104.2±22.80
*Potamogeton*	1.42±1.38	10.75±5.18	12.26±16.19	41.60±6.10

*Ceratophyllum demersum* L. is a free floating perennial macrophyte growing in slow flow and nutrients-rich water bodies. It represents an important food source for invertebrates, fish and herbivorous aquatic birds. The analysed *Potamogeton* species (*Potamogeton perfoliatus* L., *Potamogeton lucens* L., and *Potamogeton pectinatus* L.) are submerged, rooted, flowering aquatic plants that grow in alkaline waters. They form beds that represent food source and shelter for fish species, aquatic invertebrates, waterfowls etc. Both genera contribute to the establishment of the protected habitat on European level - 3150 - Natural eutrophic lakes with *Magnopotamion* and *Hydrocharition* – type vegetation [29].

The species were chosen based on their previous use as bioindicators in aquatic ecosystems and due to the fact they are one of the most frequent aquatic plant species from the Danube River ecosystems in the Iron Gates Natural Park. Metals concentrations in the four aquatic plants considered in our survey are displayed in Table 3.

When grouping the samples on plant genus (i.e. *Ceratophyllum* and *Potamogeton*) and applying the Mann–Whitney test, differences appear for all the elements (U = 214, p=0.008 for cadmium, U = 216, p=0.007 for copper, U = 223, p=0.003 for lead, U = 241, p<0.001 for zinc). *Ceratophyllum* concentrates higher amounts of elements compared to *Potamogeton* species, being a better accumulator (Table 4). Mazej and Germ [3] mention that copper and zinc distribute evenly throughout *Potamogeton nodosus*, while lead concentrate in higher amounts in the plant root. Subsequently, the differences between the two genera for copper and zinc cannot be assigned to differences in elements uptake from the water body. Fritioff et al. [19] attribute lower metal concentration to species with higher biomass such as *Potamogeton*.

Differences also appear between the four species with the exception of *Ceratophyllum demersum* vs. *Potamogeton perfoliatus*, where they are not statistically significant (Dwass-Steel-Chritchlow-Fligner test = −0.73 p=0.96 for cadmium, (Dwass-Steel-Chritchlow-Fligner test = −0.51, p=0.98 for copper, Dwass-Steel-Chritchlow-Fligner test = −0.87, p=0.93 for lead and Dwass-Steel-Chritchlow-Fligner test = −1.82, p=0.57 for zinc). Our results, expressed as mean concentration ± 1 standard deviation for each species, indicate the highest concentration of all four elements in *Ceratophyllum demersum* (see Table 3). Among the *Potamogeton* species, the highest amounts of heavy metals are recorded in *Potamogeton perfoliatus* (1.88 μg/g for Cd; 13.14 μg/g for Cu; 13.32 μg/g for Pb; 57.96 μg/g for Zn). Our data are comparable with those obtained for cadmium and copper in the same plant species by Pajevic et al. [25] on the same sector of the Danube River on the Serbian side.

We also grouped the plant samples according to the region they were collected from (with three delimited regions – Bazias, Divici, Coronini) and no differences appear between them (Kruskal-Wallis = 5.45, p=0.07 for Bazias; Kruskal-Wallis = 4.79, p=0.09 for Divici; Kruskal-Wallis = 3.22, p=0.2 for Coronini). The same conclusion was drawn for large-flow rivers by Fawzy et al. [30] for cadmium accumulation by plants (including *Ceratophyllum demersum*) from the Nile River ecosystems. In our case, the major pollution source is located near the Coronini sampling station and this is a 250 ha complex including settling ponds, sterile deposits and ores processing facilities from the former complex ores quarrying and processing plant. This had generated an increase of copper concentration in the sediments up to ten times between 1996 and 1999 (72 μg/g and 679 μg/g, respectively) [26].

The long-term contamination of a water body leads to high amounts of metals in sediments which act like a deposit of heavy metals. Depending on the physical and chemical conditions and the water body regime (flooding, drought), they release metals into the water column, making them available for the aquatic vegetation. Metals removal from water by plants is a relatively rapid process [31]. Consequently, the metals are transferred to herbivorous fish species and aquatic invertebrates, thus entering the aquatic food chains and posing an ecotoxicological risk to species on higher trophic levels.

Starting from the hypothesis of a synergetic effect of zinc level on concentration of cadmium in the plants [17], a Spearman test was applied to our set of data. The results confirm that cadmium concentration increases with zinc concentration (Spearman rho=0.40, p=0.02). Since zinc is the heavy metal with the most important concentration in the region [26,32], it might enhance cadmium uptake by plants to significant levels, even if the ambiental concentration is very low (0.002 μg/L).

Choosing the species to be used in such a survey is very important in a protected area, especially in a habitat of a Community importance. Since alien species introduction is to be avoided, the bioindicator species needs to be chosen from indigenous ones. Furthermore, they must be present on larger period of time during a year, in order to undertake exposure levels at different river regime (including extreme conditions such as drought and flooding). Nevertheless, they need to be present along the entire analysed sector, which was the case for *Trapa natans*, present, during the sampling period, only in Coronini. *Trapa natans* is also the species with the highest biomass and it is protected by the Habitats Directive [33].

At watershed scale, it is the first study on metals bioaccumulation by aquatic plants for the Romanian side of the Danube River. It allows comparison with the surveys performed for in Serbia [25,34].

### Metal bioconcentration factors for *Ceratophyllum demersum*

The uptake rate of metals by plants from their surrounding environment is better described by the bioconcentration factor. For the floating species (*Ceratophyllum demersum L.*), the bioconcentration factor was calculated as the ratio between the metal concentration in plant tissue and its concentration in water (as an average of the metal concentrations in the four sampling sites) [35].

**Figure 2:**
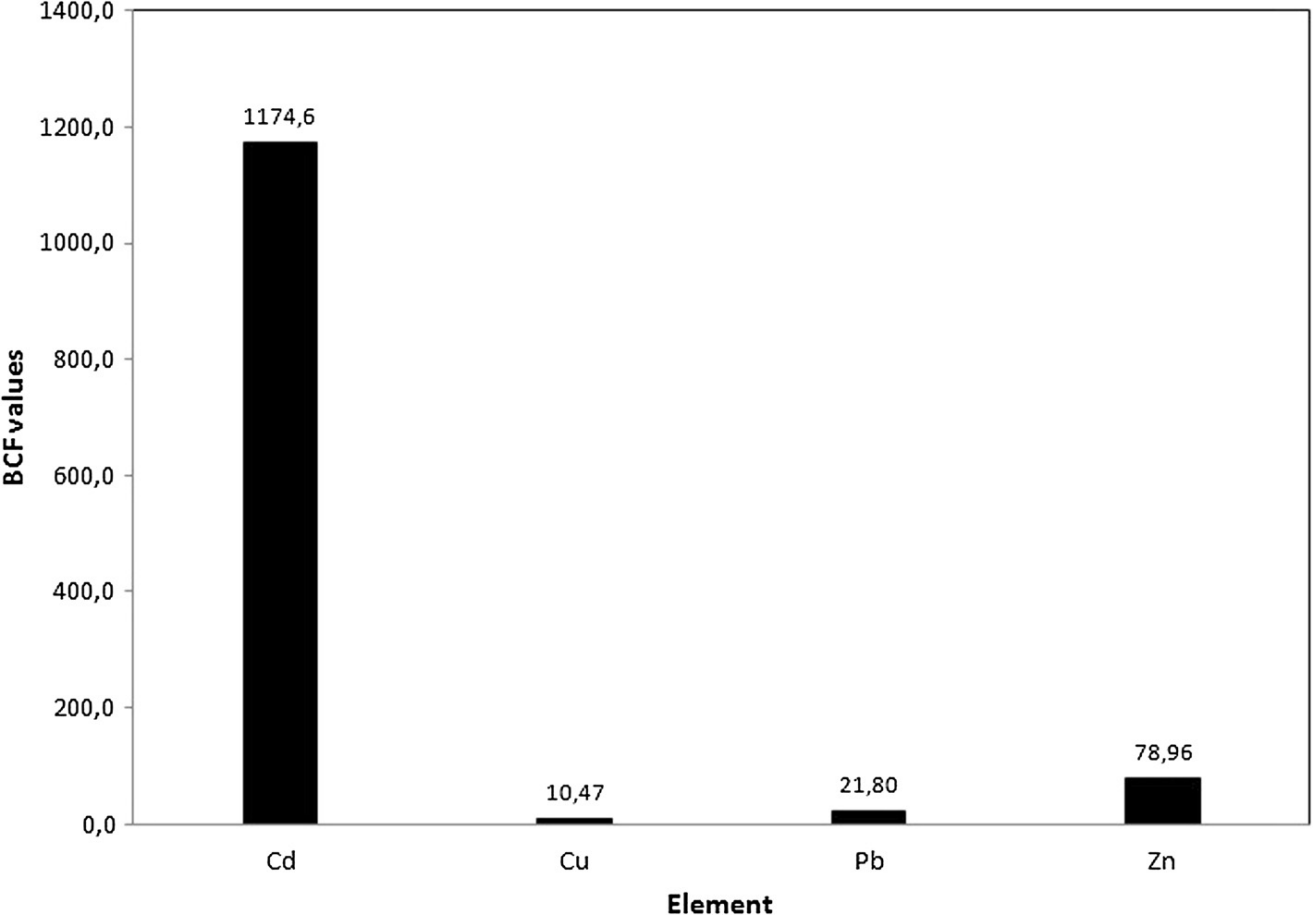
**Heavy metals BCF for *Ceratophyllum demersum *** **(values in L×g-1).**

The BCF values for *Ceratophyllum demersum* confirmed our hypothesis indicating the species as a hyperaccumulator for cadmium [14]. However, the values for the other elements are also high (see Figure 2). The sequence for the BCFs is Cd >> Zn > Pb > Cu.

The fact that the BCF value for cadmium is higher than 1000 (1174.6 L×g^-1^) or 100-fold compared on a fresh weight [36] indicates *Ceratophyllum demersum* as a suitable species for phytoremediation purposes [37].

## Conclusions

The ability to rapidly uptake the heavy metals from the environment, even at very low ambiental levels, transforms the aquatic macrophytes into efficient indicators of the aquatic ecosystems quality. Differences appear between genera – with *Ceratophyllum* showing higher metals concentrations than *Potamogeton* – and species, with *Potamogeton perfoliatus* being the most effective accumulator of trace elements.

Furthermore, *Ceratophyllum demersum* has proven to be suitable for phytoremediation processes, since its capacity to hyperaccumulate cadmium from the water body reaches values of the bioconcentration factor higher than 1000. The negative aspect of their accumulation capacity is the fact the aquatic plants represent food sources for several categories of aquatic organisms, exposing them to contamination with toxic elements.

## Competing interests

The authors declare that they have no competing interests.

## Authors’ contributions

MLM designed the survey, collected the samples during the sampling campaigns and was in charge of data interpretation and article writing. CM was in charge of the analytical measurements (including quality control) and contributed to the article by writing the methods part. LR was responsible for the statistical processing and interpretation of data. AT was in charge of sample processing. All authors read and approved the final manuscript.
